# Retraction: Enhancing ALS disease management: exploring integrated user value through online communities evidence

**DOI:** 10.3389/fneur.2026.1793079

**Published:** 2026-01-30

**Authors:** Frontiers Editorial Office

The journal retracts the article cited above.

Following publication, concerns were raised regarding the provenance of the data/materials used in this study. During the investigation, which was conducted in accordance with Frontiers' policies, the authors were unable to provide appropriate confirmation of authorisation for use of the information derived from online conversations in private social media groups. Given the concerns raised and adhering to the recommendations from UKRIO on social media research, we no longer have confidence that this work meets the consent requirements outlined in our ethics and data-use policies.

This retraction was approved by the Chief Editors of Frontiers in Neurology and the Chief Executive Editor of Frontiers. The authors agree to this retraction.

